# Enhancing Mechanical Properties and Biological Performances of Injectable Bioactive Glass by Gelatin and Chitosan for Bone Small Defect Repair

**DOI:** 10.3390/biomedicines8120616

**Published:** 2020-12-15

**Authors:** Mehri Sohrabi, Bijan Eftekhari Yekta, Hamidreza Rezaie, Mohammad Reza Naimi-Jamal, Ajay Kumar, Andrea Cochis, Marta Miola, Lia Rimondini

**Affiliations:** 1 School of Metallurgy and Materials Engineering, Iran University of Science and Technology, Tehran 1684613114, Iran; sohrabi_m@metaleng.iust.ac.ir (M.S.); hrezaie@iust.ac.ir (H.R.); 2 Department of Chemistry, Research Laboratory of Green Organic Synthesis and Polymers, Iran University of Science and Technology, Tehran 1684613114, Iran; naimi@iust.ac.ir; 3 Department of Health Sciences, Center for Translational Research on Autoimmune and Allergic Diseases–CAAD, University of Piemonte Orientale UPO, 28100 Novara, Italy; ajaykumar2420@gmail.com (A.K.); andrea.cochis@med.uniupo.it (A.C.); 4 Institute of Materials Engineering and Physics, Department of Applied Science and Technology, Politecnico di Torino, 10129 Turin, Italy; marta.miola@polito.it

**Keywords:** bioactive glass, gelatin, chitosan, 3-Glycidyloxypropyl trimethoxysilane, bone

## Abstract

Bioactive glass (BG) represents a promising biomaterial for bone healing; here injectable BG pastes biological properties were improved by the addition of gelatin or chitosan, as well as mechanical resistance was enhanced by adding 10 or 20 wt% 3-Glycidyloxypropyl trimethoxysilane (GPTMS) cross-linker. Composite pastes exhibited bioactivity as apatite formation was observed by Scanning Electron Microscopy (SEM) and X-Ray Diffraction (XRD) after 14 days immersion in simulated body fluid (SBF); moreover, polymers did not enhance degradability as weight loss was >10% after 30 days in physiological conditions. BG-gelatin-20 wt% GPTMS composites demonstrated the highest compressive strength (4.8 ± 0.5 MPa) in comparison with the bulk control paste made of 100% BG in water (1.9 ± 0.1 MPa). Cytocompatibility was demonstrated towards human mesenchymal stem cells (hMSC), osteoblasts progenitors, and endothelial cells. The presence of 20 wt% GPTMS conferred antibacterial properties thus inhibiting the joint pathogens *Staphylococcus aureus* and *Staphylococcus epidermidis* infection. Finally, hMSC osteogenesis was successfully supported in a 3D model as demonstrated by alkaline phosphatase release and osteogenic genes expression.

## 1. Introduction

The development of composites mimicking bone-like tissue in terms of physical, chemical, and topographical properties represent a fascinating challenge for tissue engineering with the aim to improve tissue healing. Accordingly, bone-dedicated biomaterials have been developed in order to be cytocompatible, absorbable, and anti-inflammatory as well as they have been molded using various topography such as block, granule, porous, and dense [1,2,3,4,5]. Between the large class of biomaterials aimed at bone repair, bioactive glass (BG) represents a very promising tool due to its ability to form apatite on the surface thus easily establishes a chemical bond with the naïve bone tissue [6,7,8]. However, despite the BG demonstrated biocompatibility, osteoconductive and osteointegrative behavior, its usage is limited in load-bearing sites. In fact, although BG holds high compressive strength, it is extremely brittle and does not withstand tensile and flexural loads [9,10,11]. Therefore, the use of polymers and cross-linkers in combination with BG improving mechanical properties can be a suitable solution. In particular, the focus of this study was devoted to developing an easy-to-handle, injectable composite paste to fill small bone defects.

Due to the need of injectability, bone pastes are viscous solutions easily handled by clinicians to fill small defects despite holding poor mechanical strengths; so, this defect opens the possibility that the paste does not support bone mechanical stress after injection thus making a new operation necessary. To overcome this limitation, we aimed to exploit the direct correlation between the mechanical properties and the composites structure; accordingly, the glycidyloxypropyl trimethoxysilane (GPTMS) was added as cross-linker to improve the BG’s mechanical strength. The hypothesized improvement is due to the crosslinking occurring between GPTMS and the paste components: in fact, the paste mechanical properties can be improved by the covalent bond between the epoxy groups of the GPTMS and the NH_3_^+^ groups in chitosan and gelatin, and by the ionic bonding with the bioactive glass powder. Previous literature already demonstrated the suitability of such strategy: for example, Kuo and Ma showed that by increasing the sodium concentration from 1 to 2 *w*/*v*, the compressive modulus of alginate hydrogels increased from 5 to 17 KPa [12]. In another study, Ishihara et al. showed that chitosan can be induced to reach the gelation phase and prevent bleeding in less than 1 min by photo-crosslinking process, thus accelerating the wound healing [13].

Bone tissue is a mixture of mineral and organic phases: so, it can be considered as a kind of composite from a chemical point of view. BG is a very promising biomaterial for bone healing, which is why it can precisely mimic the chemical structure of the naïve tissue. Here, a combination between BG and polymers has been hypothesized also to improve the biological performances of the BG thanks to the biopolymers ability to resemble the extracellular matrix (ECM) components, thus making the composites more “friendly” for cells [14]. In particular, chitosan and gelatin have been selected due to their proved cytocompatibility with different cell lineages.

Chitosan is a biopolymer derived from chitin diacetylation and because of its antibacterial, biocompatible, and biodegradable properties, it is a good candidate for biomedical applications [15,16,17,18]. The reason why chitosan can improve cell adhesion and proliferation is due to its similarity with glycosaminoglycans which are the main components of bone and cartilage ECM [19,20,21].

Gelatin is the second polymer here applied; its ability in promoting tissue repair is due to the collagen-like structure which stimulates cell adhesion providing a temporary substrate with high similarity with the cell ECM [22]. Gelatin-polysaccharide hybrids have different applications for biomedical purposes such as bioprinting, gene delivery, drug delivery, wound healing, and antimicrobial formulations [23,24]. The gelatin-polysaccharide hybrid can absorb water up to 100 times in comparison with its dry weight counterpart, thus providing suitable conditions for cell recruitment, adhesion, spread, and proliferation [25,26]. In fact, the polysaccharide component can enhance the strength of the scaffold while the Arg-Gly-Asp (RGD)-like sequence holds the ability to increase migration and cell adhesion [27,28].

Those biopolymers can be considered as very promising for tissue engineering purposes as demonstrated from the variety of gelatin-chitosan hydrogel applications such as skin, cartilage, and bone repair [29,30,31]. In previous studies, the effect of bioactive glass on polycaprolactone (PCL)/chitosan nanofibers for bone healing was investigated by Shaluman at al. [32]. In another study, Gentil et al. [33] found that bioactive glass SiO_2_-P_2_O_5_-CaO-MgO-Na_2_O-K_2_O enriched with 70 wt% gelatin-chitosan exhibited great bioactivity. Bioactivity and mechanical properties of 55S bioactive glass-chitosan composite and the influence of 55S bioactive glass on the chemical properties of gelatin-chitosan scaffolds were investigated by Peter et al. [34,35]. The effect of 45S5 bioactive glass on mesenchymal stem cell activity in chitosan-gelatin scaffolds was investigated by Xynos et al. In other study, Bielby et al. [36] and Li et al. [37] showed that 58S bioactive glass 60 mol% SiO2-33 mol% CaO-7 mol% P_2_O_5_ was biocompatible, bioactive, and biodegradable and thus suitable for bone repair applications. Maji et al. [38] prepared the 58S bioactive glass (57.44 mol% SiO_2_- 35.42 mol% CaO- 7.15 mol% P_2_O_5_)-gelatin-chitosan scaffold by the freeze-drying method and investigated the effect of adding bioactive glass to this scaffold on the mechanical properties and cellular activity of mesenchymal stem cell (hMSC).

Between the large class of bioactive glasses, the 64S can be considered a very interesting composition due to its bioactivity and rheological properties when used alone or in combination with polymers as previously demonstrated by the authors by applying a 64S glass–hyaluronic acid composition [39]. The choice of the proper glass composition is crucial as the bioactivity, injectability, mechanical, and biological properties of the composite may be influenced by the formulation of the bioactive glass. In fact, through the deposition of hydroxyapatite on its surface, bioactive glass has the potential to bond with both bone in direct contact and the surrounding soft tissues. In particular, the presence of silicon ions in the composition of the 64S glasses can be very helpful to promote osteogenesis, thus favoring the self-healing process due to their involvement in bone mineralization and proliferation of osteoblast cells [40].

Based on these premises, in this study we investigated the effects of adding chitosan and gelatin polymers on composites based on 64S bioactive glass. Moreover, the addition of glycidyloxypropyl trimethoxysilane (GPTMS) as cross-linker agent was applied to the composite in order to increase stability, enhance the mechanical properties and create a bond between the different components building the composite.

The composites were compared to the paste made of 100% BG (control) mixed with distilled water in terms of mechanical properties, degradation, apatite formation, cytocompatibility, infection prevention, and ability to support hMSC osteogenesis in a 3D-like model.

## 2. Materials and Methods

### 2.1. Bioactive Glass (BG) and BG-Polymers Composites Preparation

**Figure 1:**
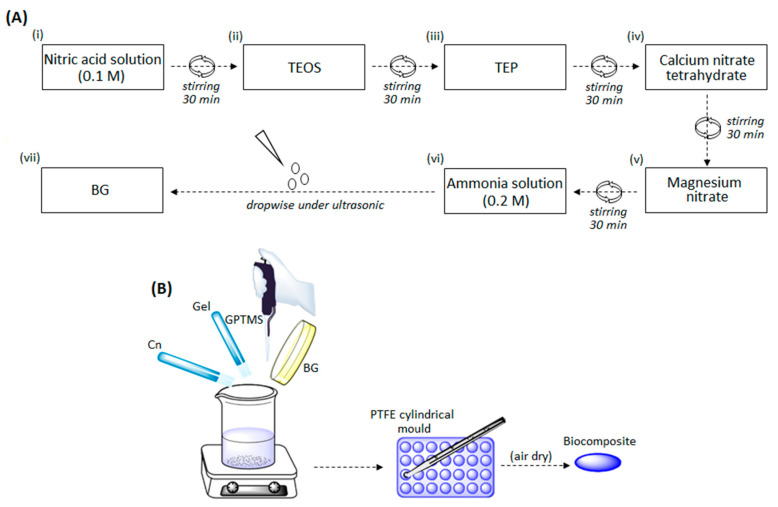
Schematization of the sol–gel process (**A**) and of the composites preparation method (**B**).

Chitosan (419419), gelatin (G9382), and 3-Glycidyloxypropyl trimethoxysilane (GPTMS) (435171) were purchased from Sigma-Aldrich, Darmstadt, Germany. Triethyl phosphate, tetraethyl orthosilicate, nitric acid solution, calcium nitrate tetrahydrate, magnesium nitrate, and ammonia solution were purchased from Merck, Darmstadt, Germany. Bioactive glass (BG) with the composition of SiO_2_ 64, CaO 27, MgO 4, and P_2_O_5_ 5 mol% was prepared by sol–gel method as shown in the Figure 1A according to previous literature [39,41].

The formed gel was incubated in the air dryer at 70 °C for 2 days and then further dried for 2 days in the oven at 120 °C. Then, to remove organic materials and nitrates, the gel was placed at 700 °C for 3 h. In the next step, the bioactive glass powder was mechanically grounded in agitation (400 rpm) for 2 h and then mixed with water to prepare the bulk 100% BG control paste. For the preparation of gelatin-chitosan-bioactive glass (Gel-Cn-BG) biocomposites, 3 wt% polymer solutions gelatin (water soluble) and chitosan (soluble in acetic acid 1M) were prepared. The composites were prepared using the same BG powder/polymers solutions with different GPTMS (10 or 20 wt% of dried polymers weight) ratios as detailed in Table 1.

According to Table 1, the materials were mixed and stirred for 30 min (room temperature). The obtained pastes (BG-Gel-Cn-GPTMS) were placed into cylindrical PTFE mold to obtain 1 × 1 cm disks and then placed at 37 °C to fully air dry as schematized in Figure 1B.

### 2.2. Physical-Chemical Characterization

#### 2.2.1. In Vitro Bioactivity Test: X-Ray Diffraction (XRD) Analysis

To evaluate composites’ bioactivity, the formation of apatite crystal was evaluated after 14 days immersion in body simulated fluid (SBF) at 37 °C, 7.4 pH, following the Kokubo’ methods [42] using a ratio of 1 g/100 mL. At the end of the time-point, the composites were removed from the SBF solution, washed twice with distilled water to remove any residual salts, and stored at 37 °C to air-dry. The phase composition of the powdered composites, before and after SBF immersion, was measured by means of XRD with Cu-Kα radiation, using a wavelength of 1.54050 Å (Dron 8, Bourevestink, Russia) at 40 kV, 40 mA, step size of 0.04, and a count time of 2 s/step.

#### 2.2.2. Mechanical Strength

To estimate the mechanical compressive strength of composites, the universal testing machine STM 120 (from Santam Co., Tehran, Iran) was used. Briefly, composites were placed at room temperature in the machine and imposed to an increasing compressive force with crosshead speed of 0.01 mm/s.

#### 2.2.3. Scanning Electron Microscope (SEM) Analysis

The morphology of the composites before and after 14 days immersion in SBF solution was visually evaluated by Scanning Electron Microscopy (SEM) (TEScan, VeGa II, Libušina, Czech).

#### 2.2.4. Degradation

To evaluate specimens’ degradation and anti-washout properties, composites, produced as described in Section 2.1, were individually weighed (=day 0) and immersed at 37 °C in SBF for 1, 7, 21, and 30 days. At each time point, the weight was recorded and compared to day 0 value; so, results were expressed as % of the day 0 value that was considered as 100% [43,44].

### 2.3. Composites’ Biological Characterization

Biological characterizations were performed using composite disks (1 cm high × 1 cm diameter). Prior to experiments specimens were sterilized under class II safety cabinet by UV light for 1 h (30 min/side) and stored at room temperature in sterile Petri dishes.

#### 2.3.1. Direct Cytocompatibility Evaluation

##### Cells Cultivation

Cells representative for the bone healing process after composite implantation were tested. Accordingly, human mesenchymal stem cells (hMSCs), human primary osteoblasts progenitors, and human endothelial cells were directly cultivated in contact with control 100% BG and test composites.

Human mesenchymal stem cells (hMSC) were purchased from Merck (C-12974 from PromoCell GmbH, Heidelberg, Germany, distributed by Merck, Milan, Italy) and cultivated in low-glucose Dulbecco’s modified Eagle Medium (DMEM, Merck) supplemented with 15% fetal bovine serum (FBS, Merck) and 1% antibiotics (penicillin/streptomycin, Merck) at 37 °C, 5% CO_2_ atmosphere. Human primary osteoblasts progenitors (hFOB 1.19 CRL-11372) were purchased from the American Type Culture Collection (ATCC, Manassas, VA, USA) and cultivated in MEM/F12 mix medium (50:50, from Sigma, Darmstadt, Germany) 10% fetal bovine serum (FBS, from Sigma) 1% antibiotics, and 3 mg/mL neomycin (G418 salt, Sigma) at 34 °C, 5% CO_2_ atmosphere. Human endothelial cells (EA.hy926, CRL-2922) were purchased from ATCC and cultivated in high-glucose Dulbecco’s modified Eagle Medium (DMEM, Sigma-Aldrich) supplemented with 10% fetal bovine serum (FBS, Sigma) and 1% antibiotics (penicillin/streptomycin) at 37 °C, 5% CO_2_ atmosphere. Cells were cultivated until 80–90% confluence, detached by trypsin-EDTA solution, harvested, and used for experiments.

##### Direct Evaluation

Cells were directly seeded onto specimen surfaces in a defined concentration (1 × 10^4^ cells/specimen), allowed to adhere for 2 h, and then submerged with 1 mL of each specific medium. Cell-seeded composites were cultivated for 1 and 3 days. At each time point, the viability of the cells in direct contact with specimens was evaluated by means of their metabolic activity by the colorimetric Alamar blue assay (Alamar Blue™, from Life Technologies, Milan, Italy) following the manufacturer’s instructions. Briefly, supernatants were removed from each well containing cells and replaced with the Alamar blue solution (10% *v*/*v* in fresh medium). Plates were incubated in the dark for 4 h at 37 °C and then 100 µL were removed, spotted into a new black 96-well plate, and fluorescence signals were evaluated with a spectrophotometer (Spark®, Tecan Trading AG, Zürich, Switzerland) using the following set-up: fluorescence excitation wavelength 570 nm, fluorescence emission reading 590 nm. Specimens made of 100% BG were used as control and considered as 100% viability; accordingly, test specimens’ values were normalized towards controls.

#### 2.3.2. Composites’ Antibacterial Properties

##### Strain Growth Conditions

Two orthopedic infection-related strain bacteria were purchased from ATCC: the Gram-positive *Staphylococcus aureus* (SA, ATCC 43300) and *Staphylococcus epidermidis* (SE, ATCC 14990) were used to assay specimens’ antibacterial properties [45]. Bacteria were cultivated onto Trypticase Soy Agar plates (TSA, Sigma-Aldrich, Darmstadt, Germany) and incubated at 37 °C until round single colonies were formed; then, 2–3 colonies were collected and spotted into 30 mL of Luria Bertani broth (LB, Merck, Darmstadt, Germany). Broth cultures were incubated overnight at 37 °C in agitation (120 rpm in an orbital shaker). Lastly, a fresh culture was prepared prior to each experiment; bacteria concentration was adjusted until 1 × 10^3^ cells/mL by diluting in fresh media until optical density of 0.00001 at 600 nm was reached as determined by spectrophotometer (Spark, Tecan, Zürich, Switzerland). Pure medium was used as blank to normalize bacteria optical density.

##### Biofilm Metabolic Activity

Sterile specimens were gently moved into a 24-multiwell plate by sterile tweezers avoiding any damages. Each specimen was submerged with 1 mL of the 1 × 10^3^ cells/mL broth culture prepared as prior described in Section 2.3.2.1; plate was incubated for 90 min in agitation (120 rpm) at 37 °C to allow the separation between adherent biofilm cells and nonadherent floating planktonic cells (separation phase) [46,47]. Afterwards, supernatants containing planktonic cells were removed and replaced with 1 mL of fresh media to cultivate surface-adhered biofilm cells (growth phase) [46,47]. Biofilm were grown at 37 °C for 1 and 3 days prior to evaluations. At each time-point, specimens were gently washed two times with phosphate buffered saline (PBS) to remove nonadherent cells and then moved to a new 24-multiwell plate where bacteria viability was evaluated by means of their metabolic activity by the Alamar blue assay as prior described for cells metabolic activity evaluation. The 100% BG paste was used as control and considered 100% viability while test values were normalized towards it.

### 2.4. Composites’ Pro-Osteogenic Properties

#### 2.4.1. 3D Bone-Like Model

After the evaluation of specimens’ cytocompatibility and antibacterial activity, the following composites were selected as the most promising and used for further analysis: b (BG-Cn-GPTMS20) and d (BG-Gel-GPTMS20). Accordingly, they were used to test cells’ ability to grow within specimens’ pores simulating a 3D matrix [48,49]. First, hMSC were mixed in a defined number (1 × 10^6^) into 50 µL of a liquid collagen matrix (PureCol™ EZ Gel solution, from Sigma); then, the collagen loading cells were slowly added dropwise into the composites at room temperature (liquid phase) to completely fill their pores. Afterwards, composites filled with cells were moved into the incubator at 37 °C to allow collagen gelation phase to act as temporary support for cells growing in a 3D matrix. The use of the collagen matrix can represent a drawback for the stem cells differentiation evaluation as it can act as a pro-osteogenic factor; however, it was necessary to maintain the cells within the pores of the scaffolds in a proper number thus avoiding cell leakage through the pores. Finally, composites were submerged with 1 mL of fresh medium and cultivated for 7 and 15 days. At each time-point cell metabolism was evaluated by the Alamar blue assay as previously described. The 100% BGs were used as control and considered 100% viability while test values were normalized towards them.

#### 2.4.2. Alkaline Phosphatase (ALP) Activity

To confirm the BG’s pro-osteogenic effect and to evaluate a possible improvement or interference coming from the polymers adjunct, the 3D model described in Section 2.4.1 was cultivated for 3, 7, and 15 days in the presence of maintenance medium (intended as low-glucose DMEM 15% FBS 1% antibiotics) thus avoiding the use of any osteogenic biochemical stimulation. At each time point supernatants were collected, and the early marker alkaline phosphatase (ALP) activity was measured to evaluate cell maturation towards bone-like lineage using a colorimetric assay (ab83369, from AbCam, Cambridge, UK). Briefly, 80 µL of supernatants were collected from each sample and mixed with 50 µL of the pNPP solution and 10 µL of the ALP enzyme (provided from the kit). After 60 min incubation, the reaction was stopped and the optical density was measured by spectrophotometer (Spark®, Tecan Trading AG, CH) using a 405 nm wavelength.

#### 2.4.3. Gene Expression

After 15 days cultivation in maintenance medium as prior described in Section 2.4.2, the expression of osteogenic genes collagen I (COL 1), alkaline phosphatase (ALP), and osteopontin (OPN) [50] was assessed by means of real-time PCR. Briefly, specimens were manually fragmented allowing cell homogenization by TRIzol reagent (Sigma). Then, RNA was isolated by isopropanol precipitation and reverse transcribed using a TaqMan kit (from Applied Biosystems, Waltham, MA, USA). For real-time PCR, TaqMan Gene Expression Assays (Applied Biosystems) were used on a GeneAmp 7500 Real Time PCR System (Applied Biosystems) using the 18S rRNA (Applied Biosystems 4310893E) as housekeeping gene. Finally, selected genes expression was normalized towards the starting expression level (intended as the seeding day expression) by the ^ΔΔ^Ct method.

#### 2.4.4. Morphological Evaluation 

Cells morphology was visually checked after 7 and 15 days culturing by means of scanning electron microscopy (SEM). Briefly, cells were fixed with 4% formaldehyde for 12 h, dehydrated by the alcohol scale (50%, 70%, 90%, and 100%, 3 h each) and finally treated with hexamethyldisilazane (from Alfa Aesar, Waltham, MA, USA) to give 3D shape to the cells. Then, specimens were mounted onto aluminum stubs using conductive carbon tape to undergo surface metallization by means of a chromium layer and observed with a SEM-EDS JEOL NEOSCOPE JCM 6000 PLUS using secondary electrons (Nikon Instrument S.p.A., Firenze, Italy).

### 2.5. Statistical Analysis of Data

Experiments were performed using three or six replicates. Normal distribution and homoscedasticity were tested with Wilk-Shapiro’s and Levene’s test, respectively. Samples were statistically compared by the SPSS software (v25, IBM, New York, NY, USA) using the one-way ANOVA test and the Tukey’s post-hoc analysis. Results were considered as significant for *p* < 0.05.

## 3. Results and Discussion

### 3.1. Composites Physical-Chemical Characterization

#### 3.1.1. XRD and SEM Analysis

**Figure 2:**
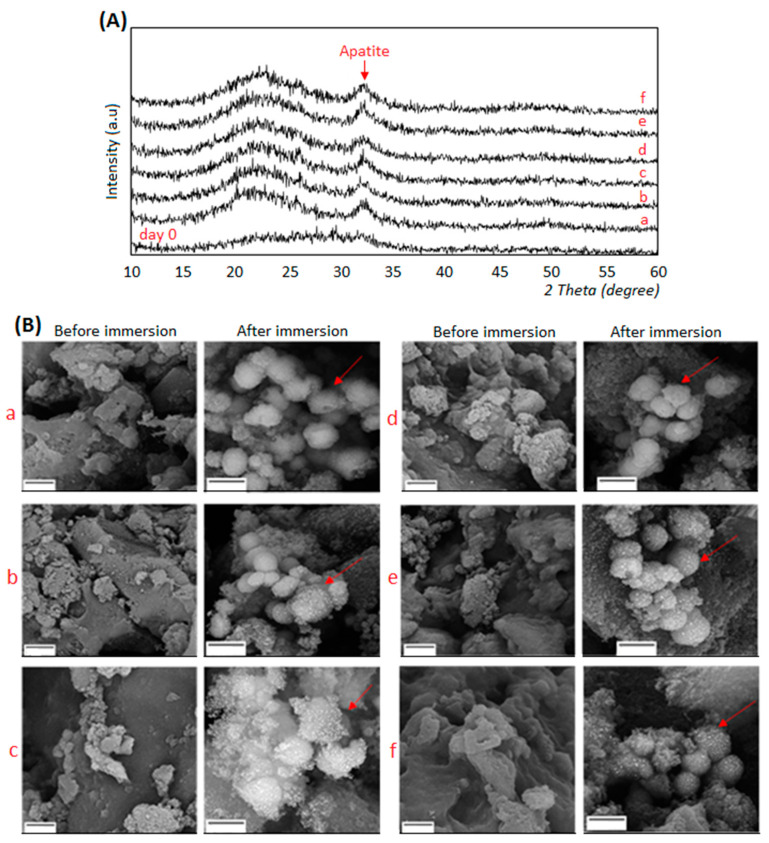
XRD spectra (**A**, upper panel) and SEM images (**B**, lower panel) of composites before (=day 0) and after immersion in SBF solution for 14 days. Apatite formation was confirmed for all the tested specimens by both XRD that showed typical apatite peaks and SEM whose images demonstrated the presence of crystals aggregates (indicated by the red arrows). SEM magnifications: 15,000× (before immersion) and 20,000× (after immersion), bar scale = 2 μm. a: BG-Cn-GPTMS10, b: BG-Cn-GPTMS20, c: BG-Gel-GPTMS10, d: BG-Gel-GPTMS20, e: BG-Cn-Gel-GPTMS10, f: BG-Cn-Gel-GPTMS20. BG=Bioactive glass, Cn=chitosan, Gel=gelatin.

The morphology and the phase analysis of composites before and after immersion in SBF solution were evaluated by XRD and SEM, respectively. Results are reported in Figure 2A,B.

XRD spectrum (Figure 2A) of composites before SBF treatment showed the presence of a broad amorphous halo, indicating that the composites were amorphous prior to SBF immersion (indicated as day 0). Conversely, after the immersion of the composites in SBF solution for 14 days, the crystalline peak of hydroxyl carbonate apatite (code: 024240) was observed at about 2θ = 32° [39,41], in addition to the amorphous halo. The formation of the hydroxyapatite layer in these composites is due to the presence of the bioactive glass (BG): in fact, the apatite formation mechanism is based on BG’s components leading to the subsequent calcium phosphate deposition [51,52]. Due to the BG’s surface reactivity, Ca^2+^, PO_4_^3−^, and Mg^2+^ ions are replaced by H^+^ ions in the SBF solution thus forming Si(OH)_4_. Then, due to the increasing dissolution, a silica-rich layer was formed on the surface of BG; afterward, PO_4_^3−^ and Ca^2+^ ions migrated through the SBF solution forming an amorphous calcium phosphate layer on the top of the glass-rich silica layer. The crystalline layer of hydroxyl carbonate apatite was then formed by the participation of OH^−^ and CO_3_^2−^ ions in the SBF solution [53]. The presence of the polymers (chitosan or gelatin) improved the bioactivity of the BG, thus enhancing the formation of apatite crystals. In fact, the negatively charged functional groups (COO^−^) of gelatin and chitosan were able to absorb calcium and phosphorus ions by electrostatic forces, thus leading to an increased apatite formation on the composite surface as previously observed by others [54,55]. Finally, SEM images (Figure 2B) offered a visual confirmation of the apatite formation onto composite surface after 14 days in SBF immersion (highlighted by the red arrows).

#### 3.1.2. Mechanical Strength

Compressive strength was applied onto control (100% BG) and GPTMS cross-linked composites to verify whether the presence of a cross-linker and the addition of polymers resulted as beneficial in relation to the ability to support mechanical stress. Results are summarized in Table 2. In general, results revealed that the chemical bond between GPTMS and other components reduces the brittleness and increased mechanical strength. In fact, specimens carrying 20 wt% GPTMS (named as b-d-f) reported the highest values if compared to the control (100% BG) and the same compositions but cross-linked with 10 wt% (intended as: a vs. b; c vs. d; e vs. f). In particular, the composites made of BG-gelatin-GPTMS wt% 20 (named as d) reported the best mechanical resistance (4.8 ± 0.5 MPa) that resulted as statistically significant in comparison with 100% BG in water bulk control (*p* < 0.05, indicated by the *). The key role of the cross-linker was confirmed by the fact that values were superior when GPTMS was used at 20 wt% in comparison to 10 wt%, thus confirming a dose-dependent effect. The enhanced mechanical properties are due to the presence and the amount of both the cross-liker and the polymers; in fact, GPTMS has the ability to bond with the amino groups of chitosan and gelatin [56,57], thus forming strong bonds conferring higher stability to the composites. Moreover, also the silanol groups in the GPTMS can bind with chitosan and gelatin so adding further stability to the composites’ structures [58]. Such results seem to be in line with previous literature; as an example, Ravarian et al. [59] demonstrated that the use of GPTMS in combination with chitosan and BG was effective in improving the composites’ mechanical properties. Accordingly, it can be speculated that the parallel increase of GPTMS wt% and mechanical properties is probably due to the presence of more covalent bonds between chitosan, gelatin, and bioactive glass.

#### 3.1.3. Degradation

**Figure 3:**
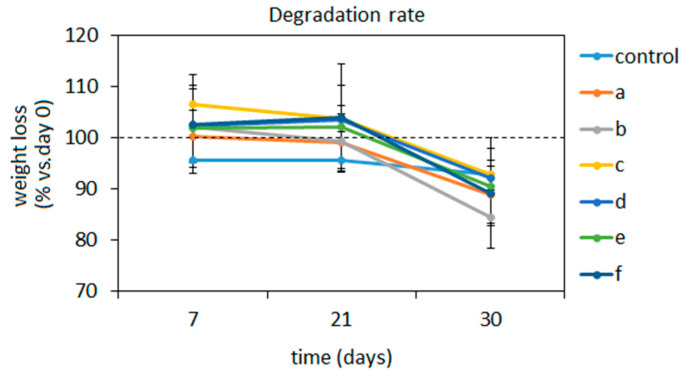
Composites’ degradation rate over time under SBF immersion at 37 °C. Results are expressed as % of the weight recorded at day 0 prior to immersion considered as 100% (indicated by the dashed line). No statistically significant differences were detected (*p* > 0.05). a: BG-Cn-GPTMS10, b: BG-Cn-GPTMS20, c: BG-Gel-GPTMS10, d: BG-Gel-GPTMS20, e: BG-Cn-Gel-GPTMS10, f: BG-Cn-Gel-GPTMS20. Experiments were done using three replicates of each composite.

Ideal composites aimed at small bone defects healing such as injectable pastes should be bioresorbable and biodegradable, but at the same time, they must be able to withstand in the injured site until the formation of the new tissue. Accordingly, the degradation of the composites was here tested over a 30 days period in physiological conditions (SBF immersion, 37 °C) by means of specimens’ weight loss in comparison with day 0 (intended as prior to SBF immersion) values that were considered as 100%. Results are reported in Figure 3.

In general, all composites demonstrated to well tolerate degradation up to day 21 (>95% in comparison to the day of weight) even if it should be mentioned that a deep analysis of ions release profile is missing in this work and it will be considered for future investigations. In the early 7 days-time point, an increase in volume was observed probably due to the fact that specimens were air- dried prior to being submerged, thus being able to shrink water up to increase their volume even if not in a significant manner in comparison to day 0 (*p* > 0.05). Then, a plateau was observed till day 21 when specimens basically maintained the same volume. Conversely, they showed degradation after 30 days with a weight loss between 16% and 10% that was not statistically significant in comparison with day 0 control (*p* > 0.05). However, this can be considered as an acceptable time for naïve bone healing so it can be hypothesized that the newly formed tissue can replace the degrading composites after 30 days [60].

The polymers adjunct enhanced composites’ strength towards degradation in comparison to 100% BG in water controls thus confirming their pivotal role for the composites stability as previously observed for the mechanical properties’ evaluation. In fact, chitosan and gelatin can swell the composite by absorbing water in their structure, then swelling increases the pore size and porosity, thereby helping to supply nutrients and oxygen to the composite network. This behavior can be of crucial importance in the healing process when cells penetrate the composite pores with the aim to repopulate the injured site. A similar trend was observed by Peter et al. [34]; they showed that the degradation of chitosan-bioactive glass scaffolds was significantly less than chitosan scaffolds thanks to mutual support. In fact, chitosan degradation is normally sped up in an acidic environment but thanks to the release of alkali groups from the bioactive glass, the environment was neutralized, and the scaffold degradation decreased [34]. Therefore, while chitosan improved the stability of the BG structure, the BG itself modified the pH of the environment thus preserving the polymer from fast degradation. 

### 3.2. Composites’ Biological Characterization

#### 3.2.1. Composite Cytocompatibility

**Figure 4:**
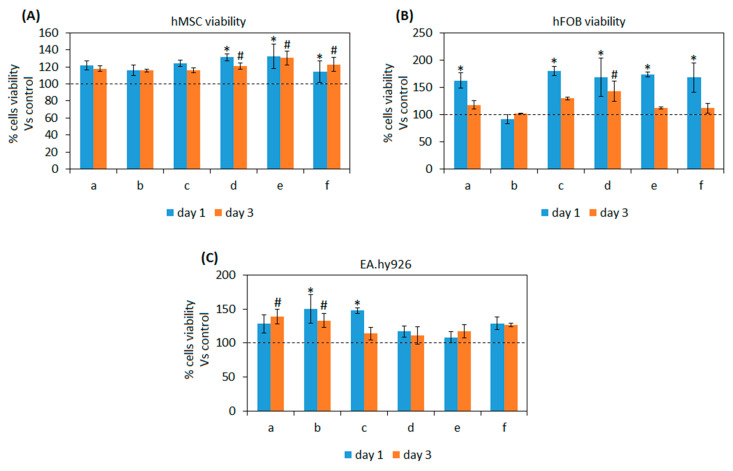
Composites’ cytocompatibility towards mesenchymal stem cells (**A**), osteoblasts progenitors (**B**), and endothelial cells (**C**). All the composites formulations reported viability values >100% (indicated by the dashed lines) in comparison with 100% BG controls. Moreover, significant differences were noticed after 1 day (*p* < 0.05, indicated by the *) or 3 days (*p* < 0.05, indicated by the #) between composites as control. Bars represent means and standard deviations. a: BG-Cn-GPTMS10, b: BG-Cn-GPTMS20, c: BG-Gel-GPTMS10, d: BG-Gel-GPTMS20, e: BG-Cn-Gel-GPTMS10, f: BG-Cn-Gel-GPTMS20. Experiments were performed using six replicates for each specimen.

As previously discussed in Section 1, the here developed composites are aimed at injectable pastes for bone small defects as temporary substitutes supporting tissue self-healing. Accordingly, materials’ cytocompatibility was in vitro assayed towards mesenchymal stem (hMSC) cells and fetal osteoblasts progenitors (hFOB): those particular cell lines were selected as representative for cells deputed for the tissue self-healing as resident progenitor cells (hFOB) or recruited from the neighborhood to migrate into the injured site (hMSC) [61]. Moreover, endothelial cells (EA.hy296) were tested in direct contact with test composites too as the neo-vascularization of the healing tissue represents a crucial step for the recruitment of nutrients as well as for the biochemical crass-talk [62]. Results are reported in Figure 4A–C.

In general, test composites demonstrated to be more cell friendly in comparison with the 100% BG controls (that were considered as 100% viability) for all the tested cells lines by reporting values >100% (indicated by the dashed lines). In detail, hMSC viability (Figure 4A) was significant in comparison with control for composites d-e-f after 1 day in direct contact (*p* < 0.05, indicated by *) as well as after 3 days (*p* < 0.05, indicated by #). Osteoblasts progenitors hFOB (Figure 4B) reported a significantly higher viability in comparison with controls after day 1 for a-c-d-e-f composites (*p* < 0.05, indicated by *) but only for d after 3 days (*p* < 0.05, indicated by #). Finally, endothelial EA.hy296 cells (Figure 4C) showed a significant increase in terms of viability in comparison with control for b-c composites after 1 day (*p* < 0.05, indicated by *) while a-b resulted significant after 3 days (*p* < 0.05, indicated by #).

According to the obtained results, it can be speculated that the introduction of polymers (gelatin or chitosan) and GPTMS as cross-linker into BG did not cause any toxic effect towards the tested cell lines; moreover, some formulations reported significantly higher values, thus suggesting to be helpful for cell adhesion and proliferation.

Those results are in line with previous literature showing that such polymers can improve osteogenesis for tissue engineering applications in combination with bioactive glasses. For example, Li et al. demonstrated that the use of gelatin doped with the bone morphogenetic protein 2 (BMP-2) was successful in promoting osteogenesis in osteoporosis [63]. Moving towards chitosan, Mokhtari et al. used chitosan-58S bioactive glass nanocomposite coatings to improve the osteogenic properties of TiO_2_ nanotubes [64].

The use of GPTMS did not introduce toxic compounds too; similarly, it was used to produce biocompatible tools such as proregenerative coatings for Ti alloys in combination with PFPE [65].

Finally, the presence of polymers and in particular of chitosan seems to promote the recruitment of endothelial cells thus probably favoring angiogenesis within scaffold pores; a similar effect was observed by Najera-Romero et al. [66] who demonstrated that heparinized chitosan/hydroxyapatite scaffolds promoted angiogenesis and ameliorated bone regeneration.

#### 3.2.2. Antibacterial Properties

**Figure 5:**
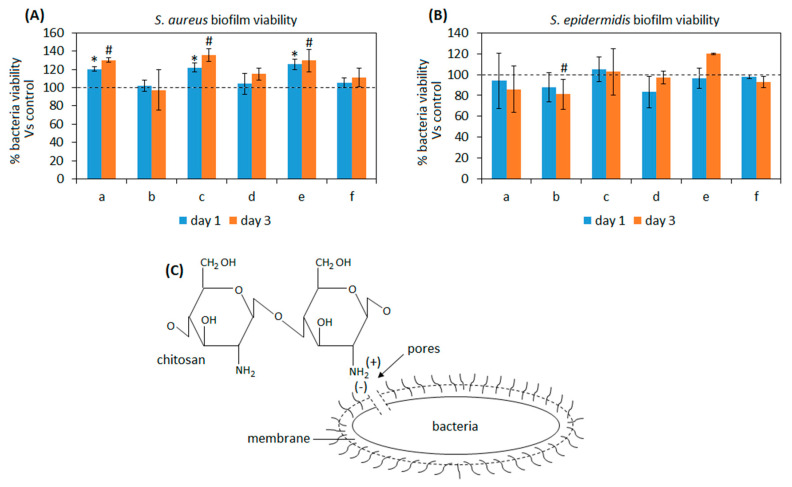
Composites’ antibacterial properties towards *Staphylococcus aureus* (**A**, SA) and *Staphylococcus epidermidis* (**B**, SE). Only the composite b (BG-chitosan-GPTMS wt20%) was successful in counteracting SA biofilm and significantly reducing SE after 3 days in comparison with bulk 100% BG in water paste controls (*p* < 0.05, indicated by #) due to chitosan properties as schematized in (**C**). On the opposite, samples a-c-e reported a higher contamination in comparison to the control (*p* < 0.05, indicated by the *) with SA infection. Bars represent means and standard deviations. a: BG-Cn-GPTMS10, b: BG-Cn-GPTMS20, c: BG-Gel-GPTMS10, d: BG-Gel-GPTMS20, e: BG-Cn-Gel-GPTMS10, f: BG-Cn-Gel-GPTMS20. Experiments were performed using six replicates for each specimen.

One of the most problematic aspects causing implant failure is represented by infections. Bacteria can enter the wound site due to the surgical procedures as well as by the injectable paste material itself if not properly sterilized [67]. Moreover, the increasing of drug-resistant pathogens strains is making infection treatment more and more difficult due to strong resistance of such bacteria to the conventional antibiotic administration. Therefore, it is of particular importance that injectable materials hold intrinsic properties to inhibit or at least to counteract bacteria colonization. Some of the here presented composites were hypothesized to hold antibacterial properties due to the presence of anti-infective compounds such as chitosan [68] and GPTMS [69]. Accordingly, we tested all the composites towards the ability to prevent the infection of *Staphylococcus aureus* (SA) and *Staphylococcus epidermidis* (SE), the two main pathogens involved in bone and joint infections. Results are reported in Figure 5.

As expected, results were strictly related to the composites formulation and in particular with the presence of chitosan. In fact, the best results were achieved by the composite b containing BG-chitosan-GPTMS wt20%: the biofilm contamination was comparable to the bulk 100% BG in water paste control materials for SA at both time points (Figure 5A, *p* > 0.05) while when SE infection was considered a significant reduction was observed after 3 days (Figure 5B, *p* < 0.05 vs. controls, indicated by #). Chitosan antibacterial properties are well known and it was demonstrated to be effective mostly towards Gram-positive bacteria but also to inhibit the biofilm formation of Gram-negative bacteria and some fungi [70]. Basically, the mechanism behind this is due to the electrostatic interaction occurring between the positive charged groups of the chitosan and the negatively charged outer membrane of bacteria that causes irreversible damage of the latter. In fact, chitosan structure is characterized by the presence of amino groups that can bind with the lipopolysaccharides and the proteins that are found within the bacteria membrane (schematized in Figure 5C); so, due to the above-mentioned interactions a change in the bacteria membrane occurs leading to the formation of pores that causes intracellular components and nutrients leaking as well as membrane lysis [71].

Referring to other composites, no evidence of antibacterial activity was observed; on the opposite, for SA (Figure 5A) a significant increase of biofilm metabolic activity was reported for specimens a-c-e after 1 (*p* < 0.05, indicated by *) and 3 days (*p* < 0.05, indicated by #) of infection in comparison with the bulk 100% BG paste. This means that probably the presence of gelatin improved the ability of bacteria to adhere and proliferate onto specimens in comparison to the bulk material. Therefore, despite a certain strain-dependent outcome and the promising results achieved from composite b (BG-Cn-GPTMS20), a more efficient strategy to improve antibacterial properties must be introduced as a future perspective to face the problem related to the infections.

#### 3.2.3. Osteogenic Properties

**Figure 6:**
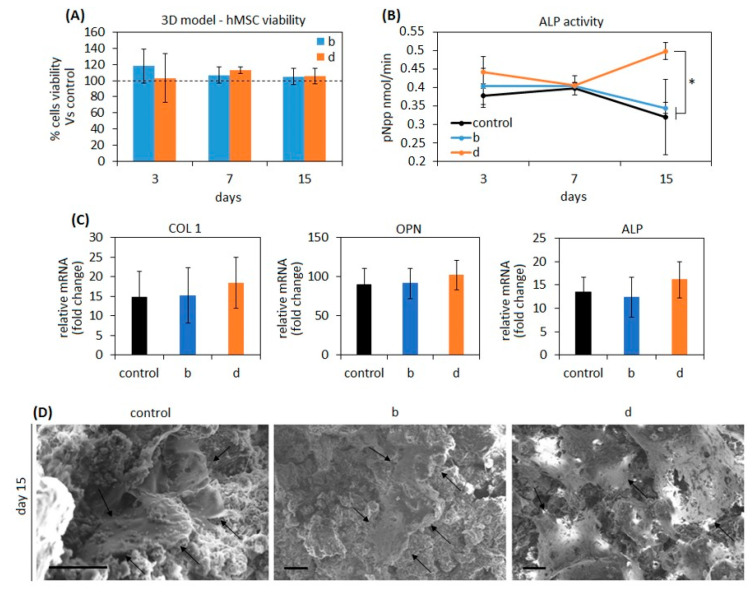
Osteogenic properties. Composite results as cytocompatible for pore 3D seeding (**A**) displaying values higher than the 100% value related to day 0 seeding (indicated by the dashed line) as well as they successfully supported hMSC osteogenic differentiation that was verified by alkaline phosphatase (ALP, **B**, * *p* < 0.05 sample d vs. b and control) increasing release in the medium and osteogenic genes collagen 1 (COL 1), osteopontin (OPN), and ALP (**C**) upregulation over the seeding day expression (fold change). Finally, SEM images (**D**) confirmed that cells grown within pores reached the osteoblast-like stellate morphology after 15 days cultivation (indicated by the arrows). SEM images: bar scale = 10 μm. a: BG-Cn-GPTMS10, b: BG-Cn-GPTMS20, c: BG-Gel-GPTMS10, d: BG-Gel-GPTMS20, e: BG-Cn-Gel-GPTMS10, f: BG-Cn-Gel-GPTMS20. Experiments were performed using six replicates for each specimen.

After cytocompatibility and antibacterial evaluations the composites b (BG-Cn-GPTMS20) and d (BG-Gel-GPTMS20) were selected for osteogenic properties assays due to the high affinity with stem cells and osteoblasts progenitors (the main populations involved in the self-healing process) and due to the ability to counteract infections of the joint pathogens SA and SE here tested. Moreover, they are representative for compositions holding or chitosan (b) or gelatin (d) in combination with BG and GPTMS (wt20%) in order to better understand a possible role of the polymers in promoting or interfering with osteogenesis over the intrinsic BG’s pro-osteogenic properties. Accordingly, mesenchymal stem cells (hMSC) were seeded into the pores of the composites and maintained in conservative medium (DMEM 15% FBS 1% antibiotics); the use of osteogenic chemicals such as dexamethasone and β-glycerophosphate was voluntarily prevented in order to exploit BG bioactivity [49] and to evaluate a possible further contribution of the polymers. This choice was also due to the need of applying collagen as temporary substrate for cell maintenance within the pores of the scaffold using static conditions; in fact, collagen can result in promoting osteogenesis, thus somehow influencing results. However, previous literature suggested that collagen stimulation is not sufficient to fully drive stem cell fate towards a complete bone-like phenotype [72,73], therefore, it can be supported that the BG’s chemistry plays the most important role defining stem cells differentiation in these experiments. Results are summarized in Figure 6.

Firstly, to confirm cytocompatibility results previously obtained by the cells monolayer experiments (showed in Figure 4A), the Alamar blue assay was used to check cells’ metabolic activity over the 15 days planned for the osteogenic evaluation. As reported in Figure 6A, the use of polymers improved cell metabolism at each time-point even if not in a significant manner (*p* > 0.05 vs. control), thus confirming composites’ cytocompatibility. Prior to running PCR to check osteogenic genes expression at day 15, the alkaline phosphatase (ALP) activity was measured in the supernatants after each medium change at 3, 7, and 15 days as an early marker of the osteogenic-like phenotype differentiation (Figure 6B); as reported in Figure 6B, similar results between 100% BG in water controls and composites b and d were obtained after 3 and 7 days (Figure 6B, *p* > 0.05) but at day 15 cultivation a trend inversion was observed for specimen d, thus suggesting a possible role for gelatin in better supporting osteogenesis in comparison with both controls and chitosan-doped specimens (Figure 6B, *p* < 0.05, indicated by *). From this point of view, a large amount of literature reported a significant contribution from gelatin in enhancing pro-osteointegrative properties; for example, Lin et al. developed gelatin-based electrospun fibrous scaffolds with enhanced ability to promote hMSC osteogenic differentiation [74]. Similarly, Wu et al. reported how the gelatin introduction into calcium silicate cements improved mechanical properties and stem cells osteogenic response [75]. A possible explanation of the gelatin contribution comes from Ren et al. [76] who showed how it can ameliorate wettability, thus facilitating cell uptake of nutrients and chemicals from the environment.

The hMSC osteogenic differentiation was checked by gene expression after 15 days cultivation by evaluating the osteogenic genes collagen type I (COL 1), alkaline phosphatase (ALP), and osteopontin (OPN). The 100% BG was considered as control due to the previously demonstrated evidence that its chemical composition is sufficient to directly promote osteogenic differentiation of mesenchymal stem cells [77,78] without the need of external further biochemical stimulation [78]. As reported by Figure 6C, a similar gene expression pattern was found, thus demonstrating that the cells seeded within composites pore underwent osteogenic differentiation in a similar manner of the BG controls. This is a confirmation that the presence of polymers did not interfere with the chemical stimulation released from the BG that stimulate cells to differentiate towards bone phenotype even if no improvements were observed due to the polymers. As previously observed for ALP activity released in the supernatant, sample d reported the highest gene fold increase expression in comparison to day 0, but results were not significant if compared to controls or sample b (*p* > 0.05).

Finally, SEM images (Figure 6D) confirmed that cells were correctly seeded into the composites’ pores interacting with surrounding environment and that they displayed the stellate morphology that is typical of the mature osteoblasts (indicated by the arrows), thus giving a visual confirmation of the bone-like differentiation occurring due to composites’ pro-osteogenic activity.

## 4. Conclusions

In order to ameliorate injectable pastes for small bone defect mechanical properties and biological performances, chitosan and gelatin were successfully coupled with bioactive glass using GPTMS as cross-linker. The composites demonstrated bioactive behavior and superior mechanical resistance towards compressive stress than pure glass as well as they improved the metabolic activity of cells deputed to undergo the tissue self-healing. Moreover, composites supported stem cells’ osteogenic differentiation in a 3D model avoiding the use of biochemical factors thus confirming strong bioactivity. However, composites demonstrated to hold poor antibacterial properties, thus failing in reducing pathogen infection which represents nowadays a primary reason of implant failure; from this point of view, more efforts should be directed in the future to conferring intrinsic antibacterial properties in order to prevent infections, as well as a proper sterilization route compatible with clinical applications, has to be achieved. Unfortunately, these limitations represent a severe hurdle for the clinical application of the composite despite the promising results obtained by the mechanical properties and bioactivity points of view.

## Figures and Tables

**Table 1:** Bioactive glass (BG) and BG-polymers chitosan (Cn) or gelatin (Gel) composites settlement.

Cn ^1^	Gel ^1^	BG ^1^	GPTMS ^2^	Specimen Code	Paste Preparation
0	0	0.25	0	Control	Mixed with distilled water
1	0	0.25	10	a	-
1	0	0.25	20	b	-
0	1	0.25	10	c	-
0	1	0.25	20	d	-
0.5	0.5	0.25	10	e	-
0.5	0.5	0.25	20	f	-

^1^ All values for polymer solutions and bioactive glass powder are expressed in grams. ^2^ All values for GPTMS are expressed as wt%.

**Table 2:** Composites’ mechanical strength evaluation.

Specimen	Control	a	b	c	d	e	f
Value ^1^	1.9 ± 0.1	1.8 ± 0.5	2.5 ± 0.2	3.7 ± 0.3	4.8 ± 0.5 *	2.8 ± 0.3	3.2 ± 0.2

^1^ Results are expressed in MPa. Experiments were done using three replicates for each specimen. a: BG-Cn-GPTMS10, b: BG-Cn-GPTMS20, c: BG-Gel-GPTMS10, d: BG-Gel-GPTMS20, e: BG-Cn-Gel-GPTMS10, f: BG-Cn-Gel-GPTMS20. *=*p* < 0.05 vs. 100% BG in water bulk control.
